# Commentary: Can the microwave auditory effect be “weaponized”?

**DOI:** 10.3389/fpubh.2022.1118762

**Published:** 2023-01-09

**Authors:** Kenneth R. Foster

**Affiliations:** Department of Bioengineering, University of Pennsylvania, Philadelphia, PA, United States

**Keywords:** infrared radiation, anomalous health incident, Havana syndrome, retinal burn, thermoacoustic sound generation

## Introduction

Since 2016 there has been considerable discussion about anomalous health incidents (AHIs), popularly called the Havana syndrome, among US personnel stationed in Havana and elsewhere. One widely discussed theory is that the incidents were the result of attacks using pulsed microwave energy resulting in nonspecific symptoms reminiscent of vestibular disturbances (1, 2). In the commented paper, Foster et al. (3) analyzed thermoacoustically (TA) induced acoustic transients produced in a simple tissue model from high intensity pulsed microwaves over a wide frequency range and concluded that microwave pulses at extreme but feasible fluences (incident energy densities per pulse) could produce physiologically significant levels of acoustic energy in the head. The possibility of attacks by microwaves was considered in detail by JASON (4), an expert group commissioned by the U.S. Department of State, which could find no evidence for the use of microwaves. The practical difficulties of beaming high peak power microwaves at subjects in a “stealthy” manner seem daunting in any event.

## Infrared TA transduction

Thermoacoustic sound generation results when a fluid is subject to rapid heating due to thermal expansion. The generation of acoustic waves is most efficient when the heating occurs over times shorter than the stress relaxation time of the medium (about 1 μs for the present case). The theory behind the effect is simple (3) and applies equally to pulsed laser light (5) as to pulsed microwaves. Unlike high peak power microwave sources, which are typically used only in classified military environments, high peak power lasers are commercially available and used for a variety of industrial applications. Such lasers are small (can be placed on a desktop) and together with their power supply and coolant system (which are typically in cabinets of < 1 m^3^ volume) and power source could be fitted into a van. Such lasers could transmit beams over long distances or, *via* fiberoptics, into enclosed spaces.

For example, the ANL10k10 Nd:YAG laser (Ekspla, a Lithuanian firm) generates 5 ns infrared pulses at a wavelength of 1.06 μm, with an output energy of 10 J/pulse and pulse repetition rate of 10 Hz. Such energy is invisible to humans. The transmission coefficient of this radiation into skin is high, and its (1/e) energy penetration depth in tissue is about 3.5 mm (6)—similar to those of 6 GHz microwaves. The laser beam would have to be expanded (to avoid skin burns from a narrow high intensity beam) and then aimed at the target, both of which are technically easy to do. This pulse energy, if uniformly distributed in a beam of 1 m^2^ area, would create a radiant exposure to a targeted individual of 10 J/m^2^.

When this energy is absorbed in skin, the resulting thermal expansion of tissue water will generate acoustic waves that will propagate deeper into tissue. The frequency range of the acoustic energy is determined by the energy penetration depth of the radiation in tissue, and the peak sound pressure is determined by the incremental amount of energy deposited in tissue, provided that the pulsewidth is shorter than the stress relaxation time of the medium. For IR wavelengths near 1 μm, IR energy is relatively highly penetrating in tissue, which results in relatively low frequency TA signals.

**Figure 1:**
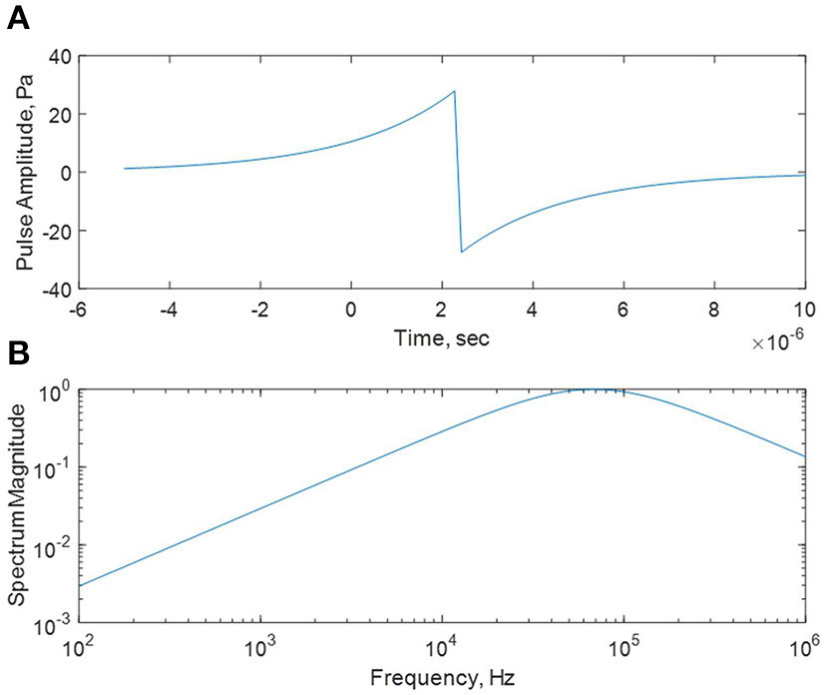
**(A)** Acoustic wave induced in a plane model of tissue by a short pulse of IR radiation with energy penetration depth in the tissue of 3.5 mm and radiant exposure of 1 J/m^2^. The pulse is assumed to be completely absorbed in the tissue and occurs at *t* = 0 in this figure. **(B)** Magnitude spectrum of the waveform. Calculated using Equations (6) and (7) of (3) assuming tissue density of 1,109 kg/m^3^ and heat capacity of 3,390 J/(kg K). For pulses shorter than the stress relaxation time (≈1 μs) the induced TA wave is essentially independent of the pulse duration.

Table 1 and Figure 1 summarize the induced acoustic energy that would be generated within the head by exposure to a single pulse of 1.06 μm laser radiation with radiant exposure of 1 J/m^2^, based on Foster et al. (3). The peak acoustic sound pressure level (SPL) is about 28 Pa (123 dB re 20 μPa), with a broad spectrum centered at 68 kHz (Figure 1B). The spectrum of the acoustic pulse is very broad, extending from the audible frequency range (e.g., ≈3 Pa or about 103 dB at 3 kHz) and upwards to hundreds of kHz. Because acoustic energy in this low-ultrasonic frequency range travels for long distances in tissue, the net acoustic stimulus in the head would consist of a series of echoes lasting for perhaps 1 ms (based on experience with microwaves), repeated at the pulse repetition rate of the laser (10 Hz for the laser described above). Curthoys et al. (8) reported thresholds for stimulation of otolithic neurons in the guinea pig cochlea of about 80 dB SPL at 3 kHz for air conducted sounds, which is a rather different stimulation than from acoustic waves that are directly generated in the head.

**Table 1:** Acoustic waves produced by a 5 ns pulse of 1.06 μm infrared energy from a commercial Nd:YAG laser incident on a fluid with acoustic properties similar to those of tissue.

**Energy penetration depth L mm**	**Pulse width (ns)**	**Assumed energy density absorbed in the head, J/m^2^**	**Incremental temperature rise at the tissue surface after each pulse (μK)**	**Peak pressure increase, Pa (dB re 20 μPa)**	**Peak acoustic frequency (kHz)**
3.5	5	1	76	28 (123)	68

Calculations based on (3).

Thus, it appears that thermally generated acoustic waves from exposure to the head to short IR pulses from commercially available lasers are in the range that could excite vestibular responses, although a more detailed analysis is clearly needed. Such laser pulses would be invisible to an exposed human being, produce negligible temperature increases in the skin (< 0.1 millidegree after each pulse) but the induced acoustic energy might be physiologically significant. However, retinal burns, a significant potential hazard of pulsed IR energy in this spectral range, would be a concern if the beam were incident on the face as opposed to the side of the head. Allen et al. (7) determined that the threshold exposure for retinal damage in the rhesus monkey from a single 4 ns pulse of Nd:YAG laser radiation is about 0.16 mJ measured at the corneal surface. This corresponds to a radiant exposure of about 6 J/m^2^; retinal damage thresholds in humans would presumably be similar. The victim might not notice a brief exposure leading to a retinal burn, but may later experience deterioration of vision. One potential use of this (entirely hypothetical) device would be for harassment, to induce frightening experiences in a subject but not cause injury. The feasibility of such a device for that purpose would depend on the difference between thresholds for auditory/vestibular stimulation and retinal injury, which at present can only be roughly estimated.

## Conclusion

This preliminary analysis suggests that, in principle, high peak power IR lasers can induce auditory/vestibular responses in humans *via* thermoelastic sound generation when directed against the head. Developing a practical non-lethal weapon would require adapting the laser and associated hardware for portable use, and adjusting the beam characteristics, power output, and wavelength to produce objectionable responses while minimizing, as far as possible, the likelihood of eye damage to the subjects. Unlike the case of high peak power microwave generators used in classified weapons programs, high peak power pulsed lasers are commercially available (but potentially are very hazardous to an untrained user) and TA sound generation in the head from pulsed IR radiation would be relatively easy to study. If reasonable suspicion exists that some individuals were exposed to such radiation, they should be examined for possible retinal injury. Non-lethal weapons of this sort are hypothetical, but seem more feasible than analogous weapons using pulsed microwaves and would potentially be of interest to governments around the world which have already made considerable investments in laser weapons.

## Author contributions

The author confirms being the sole contributor of this work and has approved it for publication.

## References

[1] NASEM. An Assessment of Illness in US Government Employees and Their Families at Overseas Embassies. Washington, DC: National Academies Press (2020). 33411434

[2] Lin JC. Sonic health attacks by pulsed microwaves in Havana revisited [health matters]. IEEE Microwave Mag. (2021) 22:71–3. 10.1109/MMM.2020.3044125

[3] Foster KR Garrett DC Ziskin MC. Can the microwave auditory effect be “weaponized”? Front Public Health. (2021) 9:788613. 10.3389/fpubh.2021.788613 35004589 PMC8733248

[4] JASON. An Analysis of Data and Hypotheses Related to the Embassy Incidents. JSR-21-01. McLean VA: The Mitre Corporation (2021).

[5] Viator JA Komadina J Svaasand LO Aguilar G Choi B Nelson JS. Comparative study of photoacoustic and reflectance methods for determination of epidermal melanin content. J Investig Dermatol. (2004) 122:1432–9. 10.1111/j.0022-202X.2004.22610.x 15175034

[6] Douplik A Saiko G Schelkanova I Tuchin VV. The Response of Tissue to Laser Light. Lasers for Medical Applications. Philadelphia PA: Woodhead Publishing (2013). p. 47–109.

[7] Allen RG Thomas SJ Harrison RF Zuclich JA Blankenstein MF. Ocular effects of pulsed Nd laser radiation: variation of threshold with pulsewidth. Health Phys. (1985) 49:685–92. 10.1097/00004032-198511000-00001 4066331

[8] Curthoys IS Vulovic V Burgess AM Sokolic L Goonetilleke SC. The response of guinea pig primary utricular and saccular irregular neurons to bone-conducted vibration (BCV) and air-conducted sound (ACS). Hear Res. (2016) 331:131–43. 10.1016/j.heares.2015.10.019 26626360

